# Does the teicoplanin skin test predict IgE-mediated hypersensitivity? 

**DOI:** 10.5414/ALX02632E

**Published:** 2026-05-21

**Authors:** Nur Betül Baştuğ İnan, Özge Göktürk, Yavuz Karahan, Kurtuluş Aksu

**Affiliations:** Division of Immunology and Allergy, Department of Chest Diseases, Ankara Atatürk Sanatoryum Training and Research Hospital, University of Health Sciences, Ankara, Türkiye

**Keywords:** teicoplanin, IgE-mediated hypersensitivity, skin prick test, intradermal test

## Abstract

Various delayed-type hypersensitivity reactions have been reported following teicoplanin administration, including drug rash with eosinophilia and systemic symptoms (DRESS syndrome), and acute generalized exanthematous pustulosis (AGEP). Until recently, IgE-mediated hypersensitivity reactions to teicoplanin were considered to be rare. However, reports of such reactions have increased in recent years. These case reports also suggest that teicoplanin skin testing can predict IgE-mediated hypersensitivity reactions. In the present case, teicoplanin skin prick and intradermal tests were negative, yet an immediate skin reaction occurred during provocation.

## Introduction 

Teicoplanin is a semi-synthetic glycopeptide antibiotic that inhibits bacterial cell wall synthesis. It is an effective agent against Gram-positive microorganisms. Teicoplanin is now used as first-line prophylactic treatment in some major surgical procedures and is often the preferred treatment for those with a penicillin allergy. It is also increasingly used in the treatment of methicillin-resistant *Staphylococcus aureus* infections [1]. Various delayed-type hypersensitivity reactions have been reported following teicoplanin administration, including maculopapular rashes, DRESS (drug rash with eosinophilia and systemic symptoms) syndrome, and AGEP (acute generalized exanthematous pustulosis) [2, 3]. IgE-mediated hypersensitivity reactions to teicoplanin were considered rare until recently. However, case reports of IgE-mediated hypersensitivity to teicoplanin have been increasing in recent years [3, 4, 5]. These case reports also suggest that teicoplanin skin testing can predict IgE-mediated hypersensitivity reactions [4, 5]. 

The present case is a case in which teicoplanin skin prick and intradermal tests were negative but an early allergic reaction developed during provocation. 

## Case report 

We present a case in which a immediate hypersensitivity reaction developed during teicoplanin provocation despite negative skin prick and intradermal tests with teicoplanin. A 66-year-old woman presented to our clinic with suspected drug allergy. She had begun using teicoplanin and paracetamol 7 days prior after experiencing pus discharge from her knee following osteoarthritis surgery. Following this treatment, non-itchy rashes developed on her body. All medications were discontinued, and intravenous methylprednisolone was initiated. She was then seen by us. On examination, mild erythematous plaques with a centripetal distribution occasionally extending to the extremities were present. The mucosa was normal, and there was no facial edema or peripheral lymphadenopathy. She had no known history of food, nutrition, or bee allergies. Her tests revealed an eosinophil count of 970/µL, a C-reactive protein of 43 mg/L, and a white blood cell count of 10,600/µL. 

When the patient was assessed at our clinic, the observed symptoms were considered consistent with a drug allergy. It was recommended that the patient’s teicoplanin intake be restricted and a different agent be selected for antibiotic therapy. However, the infectious diseases department determined that the alternative treatment was not as effective as teicoplanin and that teicoplanin should be continued. During this time, the rash from the patient’s initial reaction resolved completely. 

Drug tests with teicoplanin were planned for the patient. The patient gave written informed consent before the procedure began. A skin prick test with teicoplanin at a concentration of 20 mg/mL and an intradermal test with 2 mg/mL were performed [6]. Both tests were negative. Because the reaction that developed before the patient was referred to our clinic was consistent with an IgE-mediated early reaction, late-reaction tests were not performed. The patient was initiated on drug challenge under observation in our clinic, and a 30-minute slow infusion of teicoplanin was initiated. Within 15 minutes of starting the infusion, urticaria developed on the patient’s back. Blood pressure was 120/75 mmHg, pulse rate was 80 bpm, and oxygen saturation was 96%. No uvular edema or angioedema was detected. The teicoplanin infusion was stopped immediately, and intramuscular pheniramine and 40 mg intravenous methylprednisolone were administered. The urticaria resolved after 1 hour. Teicoplanin was discontinued, and the patient and the follow-up clinic were informed. 

## Discussion 

The probability of developing an allergic drug reaction to teicoplanin is low. Delayed-type hypersensitivity reactions, such as DRESS syndrome and AGEP, are the most commonly observed allergic reactions following teicoplanin administration. In recent years, reports of IgE-mediated hypersensitivity reactions have also begun to emerge [2, 4, 5]. Skin tests were positive in the reported cases of teicoplanin-induced anaphylaxis and thus helpful in predicting IgE-mediated hypersensitivity to the drug [4, 5]. In contrast, in the case evaluated in our clinic and presented in this paper skin prick and intradermal tests with teicoplanin were negative, although an immediate reaction, urticaria, developed during provocation with the drug within minutes. 

No validated dilution concentration is available for skin tests with teicoplanin. In the presented case, teicoplanin was used at a concentration of 20 mg/mL for the skin prick test and at a dose of 2 mg/mL for the intradermal test, which had previously been used in a number of cases before [6]. After negative skin test results were obtained, the patient underwent provocation testing with teicoplanin. A slow 30-minute teicoplanin infusion was initiated, and an itchy rash developed on the back of the waist appearing as erythematous, edematous plaques suggesting the clinical diagnosis of urticaria. 

Because the patient’s urticaria was an acute reaction that first developed when teicoplanin therapy was initiated, no other etiology of urticaria was suspected, and no additional investigations were performed. Furthermore, the absence of typical findings of red man syndrome, such as facial and chest flushing, burning sensation, and neurological symptoms, excluded that diagnosis which should be considered in the differential diagnosis, particularly when it occurs with vancomycin. 

Our case shows that teicoplanin can cause immediate skin reactions and it highlights that negative skin prick test and intradermal test are not sufficient to exclude this condition. 

## Informed consent 

Consent was obtained from the patient for this case report. 

## Authors’ contributions 

Kurtuluş Aksu: Clinical follow-up of the case, writing of the case report, literature research, approval of the final version of the article, and uploading it to the journal. 

Nur Betül Baştuğ: Clinical follow-up of the case, writing of the case report, literature research, approval of the final version of the article, and uploading it to the journal. 

Özge Göktürk: Clinical follow-up of the case, writing of the case report, literature research, approval of the final version of the article, and uploading it to the journal. 

Yavuz Karahan: Clinical follow-up of the case, writing of the case report, literature research, approval of the final version of the article, and uploading it to the journal. 

## Funding 

No funding was received for the study. 

## Conflict of interest 

All authors disclose that no conflict of interest may have influenced either the conduct or the presentation of the research. 
